# A rule‐based, feedback‐driven framework for fully automated prostate VMAT planning

**DOI:** 10.1002/acm2.70751

**Published:** 2026-08-24

**Authors:** Joel Sangster, Megan Taylor, David Jolly, Jerome Gastaldo, Friedlieb Lorenz

**Affiliations:** ^1^ Department of Radiation Oncology St George's Cancer Care Centre Christchurch Canterbury New Zealand

**Keywords:** automation, monaco, prostate

## Abstract

**Background:**

Manual prostate volumetric modulated arc therapy (VMAT) planning in Elekta Monaco is complex, time‐intensive, and prone to planner variability. Existing automation methods, such as knowledge‐based planning (KBP) and cloud‐based solutions often rely on external models rather than Monaco's native optimization. A treatment planning system (TPS)‐integrated, feedback‐driven approach could improve consistency and transparency while maintaining clinical quality.

**Purpose:**

To develop and validate a fully automated planning algorithm (APA) that actively interacts with Monaco via its application programming interface (API), mimicking expert user behavior during optimization to deliver consistent, high‐quality plans.

**Methods:**

The APA was developed as a rule‐based, fully deterministic workflow within the Monaco TPS, leveraging its native constrained and multicriterial optimization framework to enable end‐to‐end automated VMAT prostate plan generation. A retrospective, paired dosimetric study was conducted on localized prostate cancer cases. For each patient, the clinically approved manual plan was compared to an automated plan generated by the APA. The workflow reads target and organ‐at‐risk (OAR) objectives from the plan, monitors Monaco's optimization feedback—including dose‐volume histogram (DVH) metrics and cost‐function weights—and iteratively adjusts objectives. Endpoints included target coverage, OAR dose metrics, complexity, conformity, and planning time.

**Results:**

Automated plans achieved non‐inferior target coverage with a median difference in PTV V57 Gy of −0.275% (*p* = 0.116) between automated and manual plans. Automated plans produced statistically significant reductions in intermediate and low rectal doses including V40 Gy (1.62%, *p* = 0.003), V32 Gy (4.28%, *p* < 0.001), and V24 Gy (8.19%, *p* < 0.001), while high rectal doses (48–60 Gy) showed no statistically significant differences (*p* > 0.05). Similarly for the bladder, automated plans significantly reduced V48 Gy (4.07%, *p* < 0.001) and V40 Gy (2.91%, *p* < 0.001), with a modest increase in high dose bladder volume (V60 Gy +1.33%, *p* < 0.001). Mean execution time for automated plans was 38.7 ± 14.7 min per case, compared to an informally estimated manual planning time of 2 h at our institution. All automated plans met institutional clinical acceptability criteria.

**Conclusions:**

A TPS‐native, feedback‐driven automation using the Monaco Scripting API can replicate clinical planning strategies without human intervention, eliminating user variability and improving consistency. This approach offers a practical pathway for high‐quality prostate VMAT planning in routine clinical practice.

## 1 INTRODUCTION

Inverse planning for prostate volumetric modulated arc therapy (VMAT) improves conformity and organ‐at‐risk (OAR) sparing, yet remains labor‐intensive and is susceptible to planner variability.^1^, ^2^, ^3^ Automation approaches; including knowledge‐based planning (KBP), large‐language models (LLM), and scripting frameworks have emerged to standardize plan quality and reduce manual workload.^4^, ^5^, ^6^, ^7^, ^8^ Treatment planning systems (TPS) such as Eclipse (Varian Medical Systems, Inc., Palo Alto, CA) and RayStation (RaySearch Laboratories AB, Stockholm, Sweden) leverage mature scripting interfaces and established KBP frameworks such as RapidPlan to replicate clinical planning strategies.^9^, ^10^, ^11^, ^12^, ^13^, ^14^, ^15^, ^16^, ^17^, ^18^, ^19^ In contrast, automation within the Elekta Monaco (Elekta, AB, Stockholm, Sweden) environment is a relatively recent feature. Prior efforts either rely solely on static templates,^20^, ^21^ external tools,^22^, ^23^, ^24^, ^25^, ^26^ or limited use of Monaco's proprietary scripting application programming interface (API).^27^

Most previously described automated planning approaches fall into one of three categories: Template‐driven methods ^21^ apply fixed objective sets and priorities and therefore rely on manual tuning to address patient‐specific trade‐offs; KBP ^4^, ^5^ predicts achievable DVH metrics from historical datasets, but depends strongly on training data quality and institutional consistency; Scripted automation frameworks in systems such as Eclipse and RayStation or external systems such as the Radiation Planning Assistant ^9^, ^14^, ^23^, ^24^ typically encode predefined optimization sequences, with limited use of real‐time optimizer state to guide decision‐making, often reducing transparency

Boonzaier et al. ^27^ describe a Monaco‐based auto‐planning approach combining knowledge‐based modelling and multicriterial optimization to generate assistive plans, where predicted dose objectives are iteratively refined to balance organ sparing and target coverage, typically requiring subsequent manual review or adjustment.

In contrast, the strategy presented here implements a closed‐loop, feedback‐driven control system within Monaco. While the use of optimization feedback has been described previously, the present approach differs in that it explicitly interrogates Monaco‐specific internal parameters, including DVH metrics, isoeffect levels, isoconstraints, and cost‐function effective weights, and uses these quantities as control signals to iteratively modify optimization constraints during runtime in a manner analogous to an expert human planner. This approach leverages Monaco's biological cost functions and inherent multicriterial optimization framework as active control signals, rather than passive optimization tools. It also aims to reduce user variability and improve consistency while maintaining a planning time comparable to other automated approaches.^24^, ^25^, ^26^

Automation within Monaco has received comparatively little attention in the literature, despite the system's widespread clinical use, largely due to its proprietary and highly constrained scripting interface. To date, to the best of our knowledge only a single peer‐reviewed study has reported validated auto‐planning strategies using Monaco's proprietary scripting API.^27^ The present work therefore aims to address this gap by providing a detailed, end‐to‐end description and clinical validation of a reproducible Monaco‐native automated planning workflow, and by evaluating whether such an approach can generate prostate VMAT plans that are dosimetrically non‐inferior to manually generated clinical plans.

This paper details the APA's development and presents a retrospective paired dosimetric comparison of automated versus manual plans. We hypothesize that this feedback‐driven automation achieves non‐inferior target coverage while improving OAR sparing compared to manual plans.

## 2 METHODS

A retrospective paired dosimetric study was performed for a cohort of 50 patients by comparing the clinically approved manual plan to an automated plan generated by our script. The following sections outline the methodology, including: patient selection, the manual planning process, the automated planning process (including algorithm development and refinement), and plan comparisons—comprising execution time and complexity analysis, dosimetric comparisons, and physical measurements.

### 2.1 Patient selection

A cohort of the 50 most recent patients treated in our department who met all inclusion and exclusion criteria was selected for this study. Eligibility criteria included: histologically confirmed, localized, node‐negative prostate cancer; treatment with external beam radiotherapy to 60 Gy in 20 fractions; availability of an existing manual VMAT plan approved by a Radiation Oncologist; and a complete structure set containing at minimum the PTV, CTV, rectum, bladder, femoral heads, and patient contour. Patients with bowel directly contacting the prostate were excluded, as these cases require a different planning approach and a separate Monaco script. This alternative workflow is beyond the scope of the present study.

### 2.2 Manual planning process

Clinically delivered plans were generated in Elekta Monaco (v6.2.2) by experienced dosimetrists following departmental protocols. Plans typically used single‐arc VMAT geometry with 6 MV beams, optimized using Monaco's biological cost functions (gEUD‐based and quadratic overdose) and Monte Carlo (MC) dose calculation engine. Monaco's inherent two‐stage optimization process was used for all plans: Phase 1 performs fluence optimization to determine the ideal intensity pattern that best satisfies the target and OAR objectives, while Phase 2 converts this fluence into a fully deliverable VMAT solution by generating and refining control points under machine constraints, preserving the dosimetry achieved in Phase 1 as closely as possible. This separation allows the optimizer to first establish an ideal dosimetric solution without delivery constraints, before restoring deliverability while preserving as much of the optimized dose distribution as possible. In Monaco, optimization objectives are specified using isoeffects and isoconstraints. The isoeffect represents the dose level toward which the optimizer actively drives a given objective, while the isoconstraint defines an upper or lower boundary beyond which objective violations are increasingly penalized. When enabled, Monaco's multicriterial optimization (MCO) option applies an internal optimization strategy whereby the system iteratively adjusts isoeffect levels for competing objectives, while keeping the user‐defined isoconstraints unchanged to automatically explore improved trade‐offs between target coverage and OAR sparing. This process proceeds without user interaction and terminates when further isoeffect reduction leads to unacceptable degradation of target coverage.

Planners had full access to Monaco's standard optimization tools, including biological cost functions, isoeffect–isoconstraint modeling, and MCO, and were free to adjust objectives and priorities according to clinical judgment. Manual planning commenced from a standardized prostate VMAT template which specified prescription, beam geometry, and initial objective sets. Planners then performed iterative optimization cycles, adjusting cost‐functions, isoeffect levels, and selected objective priorities to achieve acceptable target coverage and OAR sparing. MCO could be enabled at the planner's discretion during Phase 1 optimization, but no fixed sequence or required number of MCO runs was prescribed.

Decisions regarding when to terminate optimization and accept a plan were based on clinical experience, perceived plan quality, and workflow considerations, provided that all mandatory institutional constraints according to Table 1 were satisfied and the plan was deemed clinically acceptable by the treating physician. As a result, manual plans reflect real‐world clinical practice, including variation in number of optimization runs, objective trade‐offs, and planner‐specific stopping points.

**TABLE 1:** Institutional standards for planning objectives and dose constraints for the 60 Gy/20‐fraction prostate protocols.

Institutional dose constraints for the 60 Gy/20‐fraction prostate protocols.			
Organ	Dose (Gy)	Optimal constraint	Mandatory constraint
CTV	D2 %	64.2 Gy	—
	V60Gy	—	95 %
PTV	V57Gy	—	95 %
Rectum	V60Gy	1 %	3 %
	V56Gy	—	15 %
	V52Gy	—	30 %
	V48Gy	27 %	35 %
	V40Gy	38 %	50 %
	V32Gy	51 %	65 %
	V24Gy	70 %	80 %
	Dmean	35 Gy	—
Bladder	V60Gy	5 %	35 %
	V48Gy	25 %	50 %
	V40Gy	50 %	—
Femur L/R	V40Gy	5 %	50 %

To accelerate the optimization process, Monaco's inherent Phase 1 and Phase 2 optimizations are initially performed using a coarser dose calculation grid. Once a satisfactory dose distribution is achieved at the end of Phase 2, the grid is switched to a finer resolution and the dose is recalculated only, while segment shapes and control points remain unchanged. This final step therefore constitutes a dose recalculation rather than a re‐optimization.

### 2.3 Automated planning process

#### 2.3.1 Algorithm development

##### 2.3.1.1 Monaco template

**FIGURE 1:**
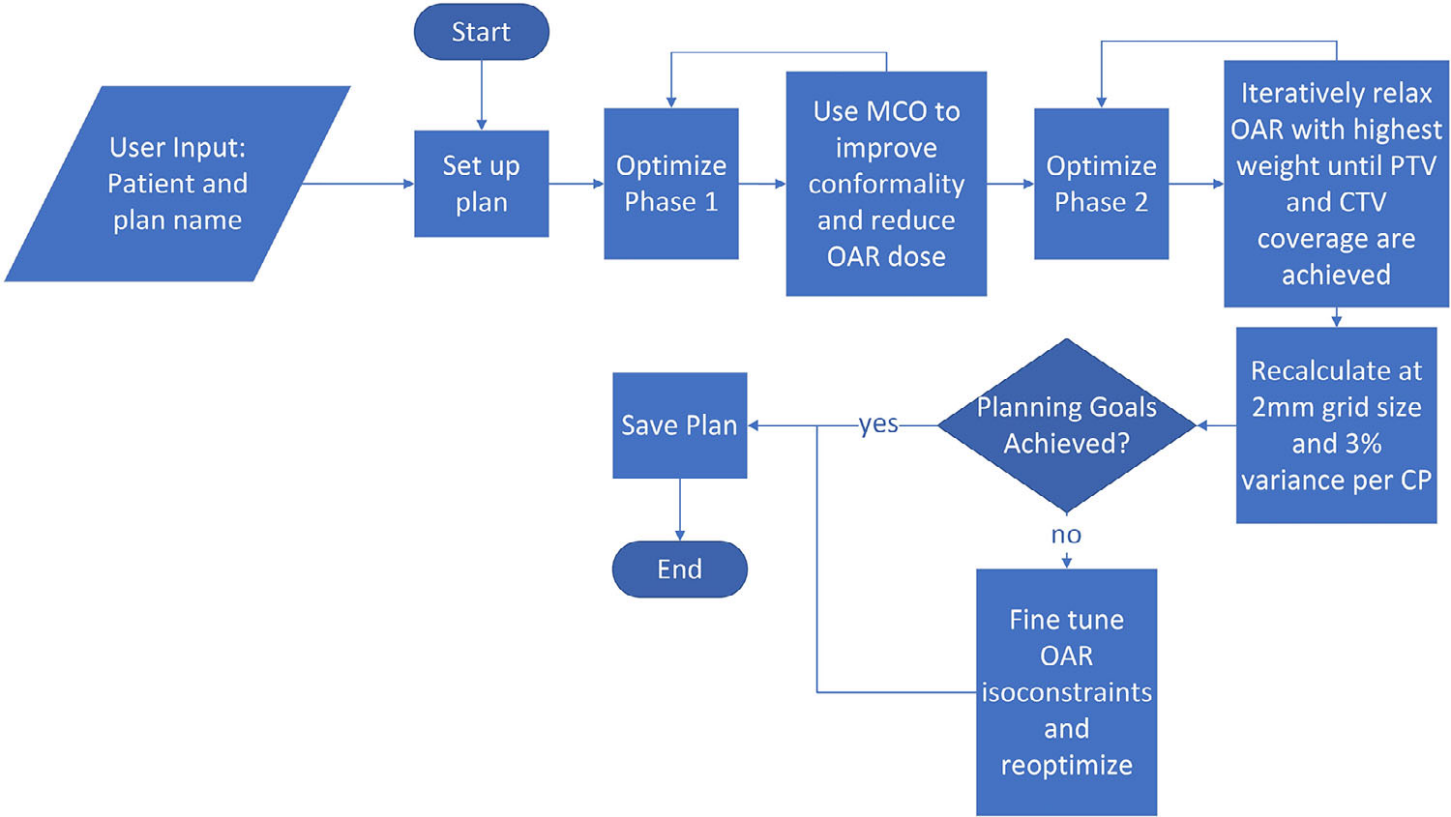
Simplified flowchart of the APA. After user initialization, multiple Phase 1 MCO optimizations are performed to improve plan conformality and reduce OAR dose. Monaco's Phase 2 optimization is then performed repeatedly while iteratively relaxing the highest weighted OAR constraint until PTV and CTV coverage are achieved. Plans are recalculated at a 2 mm grid resolution and evaluated against clinical planning goals, with further isoconstraint tuning performed as needed before final plan acceptance.

Automated treatment plans commenced from a standardized Monaco prostate template. The template encoded the full baseline configuration required for plan generation prior to any automated adaptation. This included prescription definitions (60 Gy in 20 fractions), beam geometry (one VMAT beam with 360 degree rotation) and arc configuration (arc increment of 30 degrees, collimator angle of 10 degrees), dynamic sequencing and control‐point parameters (8 SSO loops, 1 arc, 140 control points per arc, 1.0 cm minimum segment width, high fluence smoothing), initial optimization grid settings (0.49 cm), Monte Carlo dose calculation parameters (dose‐to‐medium calculation, 5% variance per control point), and a comprehensive library of preconfigured cost functions as shown in Table 2. The template incorporated serial and quadratic overdose objectives for the rectum, bladder, penile bulb, and patient structure. These initial values were identical for all patients. This baseline Monaco template is not designed to produce clinically acceptable plans in isolation; its objective settings are intentionally loose to allow MCO and subsequent feedback‐driven refinement to perform meaningful optimization, consistent with routine clinical planning workflows. The role of the APA was therefore not to define planning intent, but rather to iteratively tune the tightness of these objectives in response to patient‐specific anatomy and optimization feedback. A complete, step‐by‐step specification of the algorithm—including all numerical thresholds, iteration limits, and decision criteria—is provided as pseudocode in the supplementary material. A high‐level overview of the APA, including the role of this template as the algorithm entry point, is illustrated in the simplified flowchart shown in Figure 1.

**TABLE 2:** Monaco template used for automated plans with initial cost function parameters.

Monaco template for automated plans				
Structure	Cost function	Reference dose (Gy)	Shrink margin (cm)	Iso‐constraint
CTV	Target penalty			60
	Underdose DVH	60		96.5
PTV	Target penalty			57
	Quadratic overdose (including CTV)	61.4		0.25
	Underdose DVH	57		98
	Quadratic overdose (excluding CTV)	62.4		0.13
OptRectum	Serial (k = 16)		1.0	40
	Quadratic overdose	59		0.12
Bladder	Serial (k = 10)		0.5	50
	Quadratic overdose	60		0.3
PenileBulb	Serial (k = 3)		0.1	50
Patient	Quadratic overdose	57		1
	Quadratic overdose	40	1.0	2
	Quadratic overdose	24	3.0	3
	Maximum dose		6.0	30
	Maximum dose			63.9

##### 2.3.1.2 Implementation environment

The APA was implemented as a deterministic, rule‐based iterative optimization workflow using Monaco Scripting (v6.2.2). All plans were generated in Monaco, which was hosted on a remote HP ProLiant DL380 G10 server equipped with an Intel® Xeon® Gold 6132 CPU (2.60 GHz) and 128 GB RAM. This configuration ensured consistent execution conditions and eliminated operator‐dependent variation.

##### 2.3.1.3 Plan setup and preprocessing

Following user initialization and plan naming, the script interrogated the imported structure set to verify structural integrity and nomenclature consistency. Limited patient‐specific preprocessing was performed, including identification of small bladder volumes, which triggered predefined modifications to optimization behaviour (e.g., constraint prioritization); full decision logic is provided in the supplementary material.

##### 2.3.1.4 Phase 1 optimization and multi‐criteria optimization

After plan setup, Monaco's intrinsic Phase 1 optimization was initiated. To enhance conformality and OAR protection without sacrificing target coverage, the APA invoked a sequence of MCO runs. In Monaco, MCO is implemented as a native optimizer mode that can be enabled or disabled via a binary option. Unlike MCO frameworks in some other TPS, Monaco does not require the user to define explicit OAR–target trade‐offs, preference structures, or Pareto navigation parameters. When enabled, Monaco's MCO internally adjusts isoeffect levels for existing competing cost functions while preserving the user‐defined isoconstraints, allowing the optimizer to explore alternative solutions within the predefined objective. In this study, MCO was used solely by enabling the Monaco MCO option within Phase 1 optimization.

MCO was applied sequentially to predefined cost functions (patient, rectum, and bladder). After each run, isoeffect values were extracted and corresponding isoconstraints updated using predefined rules (see Table 3 and supplementary material).

**TABLE 3:** Isoconstraint modification rules after Monaco's MCO (IC = isoconstraint, IE = isoeffect after MCO run, QO = quadratic overdose cost function). For each objective placed under MCO control, Phase 1 optimization was repeated, after which the script interrogated the Monaco‐reported isoeffect. The corresponding isoconstraint was then modified according to rule‐based scaling factor.

Isoconstraint modification rules after Monaco's MCO		
Cost function	Condition	Modification
Patient QO 57 Gy	IE < 0.025	IC = 0.04
	IE ≥ 0.025	IC = 1.5 × IE
Patient QO 40 Gy	IE < 0.04	IC = 0.1
	IE ≥ 0.04	IC = 2.5 × IE
Patient QO 24 Gy	IE < 0.5	IC = 1.3 × IE
	IE ≥ 0.5	IC = 1.05 × IE
Rectum serial	N/A	IC = IE + 5.2
Bladder serial	N/A	IC = IE + 6

Once a given objective had been tuned, MCO control was disabled and the algorithm proceeded to the next objective.

At the end of this process, target coverage metrics were evaluated. These metrics were used to determine whether the optimization grid resolution was adequate. Optimization was initialized at a grid size of 0.49 cm; if post–Phase 1 target coverage criteria were not met, the grid was iteratively refined to finer resolutions according to predefined thresholds (fully specified in the supplementary material) until coverage was achieved.

##### 2.3.1.5 Phase 2 optimization and constraint relaxation

Following completion of all Phase 1 and MCO‐driven optimization steps, Monaco's Phase 2 optimization was initiated. Owing to the constrained nature of Monaco's optimization framework, the initial Phase 2 solution predictably favored OAR compliance at the expense of target coverage. As a result, direct satisfaction of PTV and CTV coverage objectives was not expected at this stage. To address this imbalance, the APA implemented a structured, iterative constraint‐relaxation strategy designed to selectively relax the OAR constraint limiting target coverage. During Phase 2, the highest‐weighted limiting OAR constraint was iteratively identified and relaxed according to predefined scaling factors until target coverage criteria were met or stopping conditions were reached (see supplementary material for more details).

This strategy was chosen because, in Monaco, higher effective weights indicate increased dominance of a cost function within the optimization framework, thereby reducing the optimizer's ability to simultaneously satisfy competing objectives, including target coverage. Relaxing a lower‐weighted OAR constraint would therefore have minimal impact on restoring coverage, whereas selective relaxation of the highest‐weighted constraint directly rebalances the optimization. Following each relaxation step, target coverage metrics for the PTV and CTV were re‐evaluated. This loop continued until all coverage criteria were met or the number of iterations exceeded 10.

##### 2.3.1.6 Final dose calculation

Once all target coverage objectives and OAR dose constraints were met within the optimization framework, the plan entered the final dose calculation stage. At this point, the dose calculation engine was reset and the calculation grid was set to 2 mm resolution, with a Monte Carlo statistical uncertainty of 3% per control point. The final dose distribution was then computed using Monaco's Monte Carlo dose engine. Following completion of the final dose calculation, all target coverage metrics and OAR dose objectives were re‐evaluated. In cases where the finer grid resolution or reduced statistical variance resulted in degraded target coverage or unacceptable increases in OAR dose, the APA invoked a single corrective fine‐tuning step. This step consisted of executing Phase 1 and Phase 2 optimizations sequentially using the final grid and Monte Carlo settings. At this stage, isoconstraints were intentionally kept largely unchanged to preserve the convergence achieved during earlier iterations. The only permitted adjustment was a limited modification of the rectum quadratic overdose cost function if the Rectum V60Gy metric exceeded the predefined 1% tolerance. This step is displayed as ‘Fine tune OAR isoconstraints and reoptimize’ in Figure 1. Upon successful validation of all dosimetric criteria, dose–volume histograms (DVHs) were automatically exported for both the automated plan and the corresponding clinical reference plan. Key plan characteristics, including total monitor units (MU) and end‐to‐end execution time, were recorded for subsequent analysis and comparison.

#### 2.3.2 Algorithm refinement

The APA was developed through an iterative, feedback‐driven process to ensure consistent plan quality and clinical robustness. Initial development was performed using a set of 12 representative prostate cases. This initial version of the algorithm was then reviewed by expert dosimetrists, who provided structured feedback for an additional 8 cases, detailing modifications and preferences. This feedback was used to adapt and align the algorithm with departmental planning practices. For final validation, a separate set of 50 automated plans were generated, and these 50 plans constitute the dataset presented in this study.

### 2.4 Execution time and complexity

Analysis Execution time for automated plans was measured using a built‐in timing function within the APA's code, and is presented as the mean ± one sigma standard deviation across the cohort. Complexity of the manual and automated plans for each patient was evaluated using the total MU per plan, and via calculation of the Modulation Complexity Score (MCS).^28^, ^29^, ^30^ This metric combines Leaf Sequence Variability (LSV)—a metric of variability in consecutive leaf positions and indicative of shape complexity in segments—and Aperture Area Variability (AAV)—an indicator of segment size. A lower MCS indicates a higher degree of modulation, and thus complexity. The metric is calculated per segment and weighted by segment MU to get a single value per plan. Plan data was extracted and metrics computed using an in‐house developed Python script. MU and MCS values are reported using the median and IQR.

Dosimetric Comparison For each patient, DVH metrics were extracted from both the clinically approved plan and the corresponding automated plan. The comparison included all mandatory and optimal institutional constraints for the PTV, CTV, rectum, bladder, and femoral heads, as outlined in Table 1. Target coverage was evaluated using PTV V57 Gy, and dose conformity was assessed using the Conformity Index (CI) as reported directly by the Monaco TPS, with results presented as the mean ± one sigma standard deviation. In Monaco, the CI is defined as

$$\frac{{V_{R_{x}}}^{2}}{TV\times V_{RI}},$$

Where V_Rx_ is the volume of the target receiving at least the prescription dose, TV is the total target volume, and V_RI_ is the total volume encompassed by the reference isodose, which was chosen to be 56 Gy in our case.^31^ OAR dosimetry was assessed using standard volumetric DVH parameters, including rectum V24–60 Gy, bladder V40–60 Gy, and femoral head V40 Gy. Mean rectum dose was also recorded. All DVH values were exported numerically to enable direct pairwise comparison between automated and manual plans. For visual comparison, DVHs were aggregated across the cohort to produce median curves and interquartile ranges (IQRs). Automated and manual plan metrics were compared using paired statistical tests. Normality of the distributions of paired differences was assessed using the Shapiro–Wilk test. As most DVH metrics were not normally distributed, differences between automated and clinical plans were evaluated using the Wilcoxon signed rank test. For each parameter, the median difference between plans (automated‐manual) and the associated p‐value were reported. Due to the large number of DVH metrics being compared, the p‐values were adjusted using the Benjamini‐Hochberg method to control false positive results.^32^ A significance threshold of *p* < 0.05 was used to determine statistical significance. All analyses were performed using Python through the SciPy statistical library.^33^ All graphics including bar charts, boxplots, and DVH plots were generated using Matplotlib.^34^

### 2.5 Physical measurement

A random subset of 10 out of the 50 (20%) automated plans was selected for physical measurement verification on an Elekta Synergy linear accelerator. Each selected plan was delivered to the ArcCHECK ® (Sun Nuclear) helical diode array, and the measured dose distribution was compared to the Monaco calculated dose. Gamma analysis was performed using 3%/2 mm global criteria with a 10% low dose threshold, following methodologies and tolerance levels recommended by AAPM TG218. TG218 emphasizes the use of 3D measurement systems such as ArcCHECK for VMAT QA and supports the adoption of more stringent spatial tolerances, including 2 mm distance‐to‐agreement to improve error sensitivity.^35^ Per institutional policy—and in alignment with TG218's recommended tolerance—a ≥ 95% pass rate at 3%/2 mm gamma was required for plan acceptance.

## 3 RESULTS

### 3.1 Execution time and complexity analysis

**FIGURE 2:**
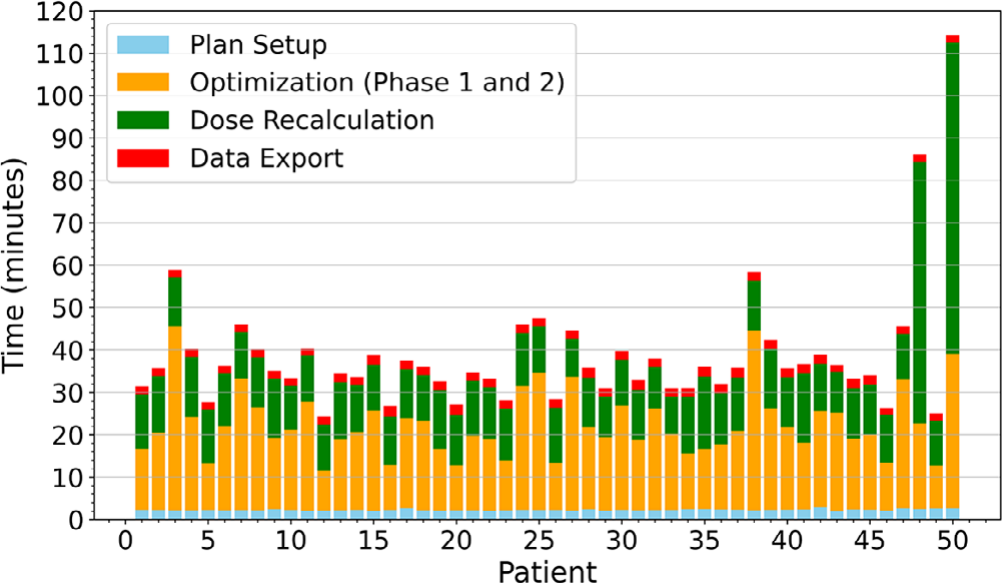
Automated planning execution time for each patient. Execution times are broken down into Plan Setup, Optimization, Dose Calculation, and Data Export.

**FIGURE 3:**
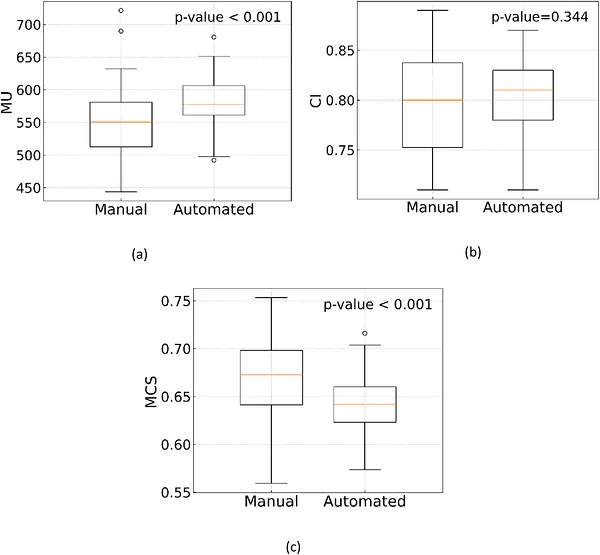
Box plots of the distribution of (a) Monitor Units (MU), (b) Conformity Index (CI), and (c) Modulation Complexity Score (MCS) across the manual and automated plans, with associated p‐values. Automated plans demonstrated a statistically significant increase in MU and a decrease in MCS, and no statistically significant differences in CI.

The mean execution time for automated plans was 38.7 ± 14.7 min. Due to the retrospective nature of the study, execution time was not measured for the clinically accepted manual plans. However, total manual planning time was informally estimated to be approximately 2 h. Figure 2 presents the execution times for the automated plans, broken down into the individual components of the workflow: plan setup, optimization (Phase 1 and Phase 2, including all cost function adjustments), dose recalculation after updating the calculation grid to 2 mm and setting the statistical variance to 3% per plan; and finally, the time required for data export. The MU per plan were higher by a modest but statistically significant margin in the automated plans compared to the manual plans as seen in Figure 3a. The median (IQR) MU values were 577.8 (45.1) for automated plans and 550.6 (68.4) for manual plans (*p* < 0.001).

Similarly, MCS were marginally lower in the automated plans, with median (IQR) MCS of 0.642 (0.037) for automated plans and 0.673 (0.057) for manual plans (*p* < 0.001). Figure 3c presents box plots of the distribution of MCS for both manual and automated plans. Automated plans demonstrated a narrower distribution of Modulation Complexity Scores compared with manual plans.

### 3.2 Dosimetric comparison

Table 4 presents the median and IQR for each dose metric across the 50 automated plans, together with the median difference relative to the manual plans and the corresponding adjusted p‐values.

**TABLE 4:** Median and IQR for each dose metric across the 50 automated plans, and median differences relative to the manual plans with corresponding adjusted p‐values. For OARs, a negative number indicates improved sparing, and for targets a positive number indicates improved coverage.

Median dose metrics for automated and manual plans							
Structure	Dose	Median (Auto)	IQR (Auto)	Median (Manual)	IQR (Manual)	Median Δ (Auto‐Manual)	Adjusted *p*‐value
CTV	D2%	63.05 Gy	0.18 Gy	62.94 Gy	0.56 Gy	0.101 Gy	0.323
	V60Gy	99.90%	0.36%	98.98%	1.28%	0.68%	<0.001
PTV	V57Gy	98.23%	0.65%	98.35%	0.77%	−0.28%	0.116
Rectum	V60Gy	0.35%	0.29%	0.36%	0.38%	−0.04%	0.901
	V56Gy	6.52%	3.25%	6.61%	3.55%	−0.09%	0.676
	V52Gy	11.37%	5.11%	11.32%	5.25%	−0.16%	0.376
	V48Gy	14.74%	6.87%	14.96%	6.01%	−0.52%	0.143
	V40Gy	20.01%	8.69%	22.03%	8.58%	−1.60%	0.003
	V32Gy	26.13%	11.83%	31.97%	10.39%	−4.28%	<0.001
	V24Gy	37.50%	13.78%	47.26%	13.99%	−8.19%	<0.001
	Dmean	19.37 Gy	2.14 Gy	19.88 Gy	1.89 Gy	−0.485 Gy	<0.001
Bladder	V60Gy	2.61%	1.31%	0.82%	1.03%	1.33%	<0.001
	V48Gy	10.40%	6.38%	14.12%	6.17%	−4.07%	<0.001
	V40Gy	14.59%	8.08%	19.03%	7.89%	−2.91%	<0.001
Femur L/R	V40Gy	0%	0%	0%	0%	0%	—

**FIGURE 4:**
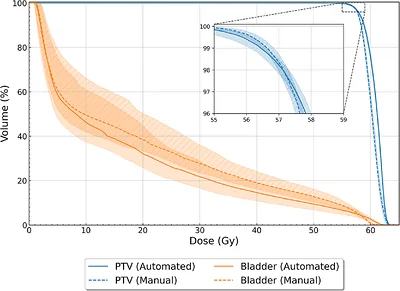
Median PTV and Bladder DVHs for the automated plan (solid line) and the manual plan (dotted line). The solid and cross‐hatched shaded regions represent the interquartile range (IQR) of the DVHs for the automated plans and clinical plans respectively. The inset plot shows the shoulder region of the PTV DVH, where the manual plans achieve marginally higher volume at 57 Gy, before falling more steeply.

All automated plans met the institutional mandatory requirement of PTV V57 Gy ≥ 95% and achieved coverage equivalent to manual plans, with a median difference in V57 Gy of −0.275% (*p* = 0.116). Figure 4 shows the median and IQR of the PTV DVHs for the automated (solid line) and manual (dashed line) plans. As illustrated in the inset plot of Figure 4, the DVHs showed a consistent crossover pattern in the shoulder region, with marginally higher volume at 57 Gy in the manual plans, before falling more steeply. In contrast, automated plans demonstrated a rightward shift beyond approximately 57 Gy, indicating a slight increase in high dose volume. This was reflected quantitatively by a small but statistically significant increase in CTV V60 Gy for automated plans with a median difference of +0.682% (*p* < 0.001), although no statistically

**FIGURE 5:**
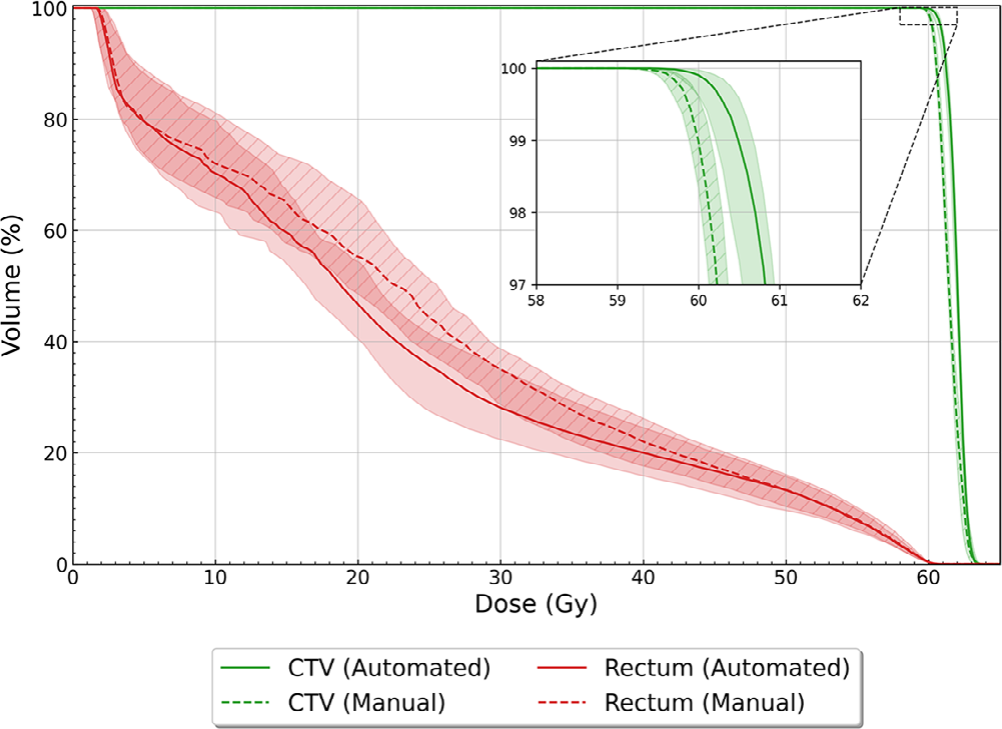
Median CTV and Rectum DVHs for the automated plan (solid lines) and the clinical plan (dotted lines). The solid and cross‐hatched shaded regions represent the inter‐quartile range (IQR) of the DVHs for the automated plans and clinical plans respectively.

Significant difference was observed in CTV D2% (+0.101 Gy, *p* = 0.323). Figure 5 shows the median and IQR of the CTV DVHs for the automated and manual plans. Across the cohort, the median (IQR) CI was 0.81 (0.05) for automated plans, and 0.8 (0.08) for manual plans.

Automated plans produced statistically significant reductions in intermediate and low rectal doses including V40 Gy (1.62%, *p* = 0.003), V32 Gy (4.28%, *p* < 0.001), and V24 Gy (8.19%, *p* < 0.001), while high rectal doses (48–60 Gy) showed no statistically significant differences (*p* > 0.05). The rectum mean dose was reduced by −0.485 Gy (*p* < 0.001) in the automated plans. This low‐intermediate dose reduction is illustrated in Figure 5, which shows the median and IQR of the rectum DVHs across the cohort for the automated and manual plans. Similarly for the bladder, automated plans significantly reduced V48 Gy (4.07%, *p* < 0.001) and V40 Gy (2.91%, *p* < 0.001), with a modest increase in high dose bladder volume (V60 Gy + 1.33%, *p* < 0.001).

Figure 4 illustrates the median and IQR of the bladder DVHs across the cohort for the automated and manual plans. Dose to the femoral heads showed no observable differences between automated and clinical plans, with the DVH curves nearly overlapping and V40 Gy = 0 across all cases in both the automated and manual cohorts. All automated plans met the mandatory target coverage objectives and OAR constraints listed in Table 1. In 46 of the 50 cases (92%), all optimal dose constraints were also achieved. In one case, the automated plan slightly exceeded the optimal bladder V60 Gy < 5% criterion, with a value of 5.79% compared with 3.71% for the clinically approved plan. In two additional cases, the automated plans exceeded the optimal bladder V48 Gy < 25% constraint, with values of 27.3% and 28.53%, respectively. For comparison, the corresponding manual plans yielded V48 Gy values of 32.65% and 24.46%. Notably, in one of these two cases, the automated plan demonstrated a lower bladder dose than the clinically approved plan. In a fourth case, the rectum V48 Gy, V40 Gy, and V32 Gy metrics for the automated plan exceeded the optimal thresholds listed in Table 1, although all values remained within mandatory clinical limits (automated: 30.11%, 41.79%, and 52.28%; manual: 23.31%, 32.61%, and 42.79%, respectively).

### 3.3 Physical measurements

All 10 plans selected for ArcCHECK measurement verification met—and substantially exceeded—the institutional acceptance threshold. The minimum gamma pass rate observed among the ten measured cases was 99.8%, indicating excellent agreement between measured and calculated dose.

## 4 DISCUSSION

This study evaluated a fully automated, TPS‐native prostate VMAT planning workflow designed to replicate expert planning strategies while reducing planner variability and manual workload. Using a retrospective, paired comparison with clinically approved manual plans, we assessed whether this feedback‐driven approach could achieve comparable dosimetric quality, consistent plan complexity, and practical clinical deliverability. The primary objective of this study was not to demonstrate superiority of automated planning over manual planning, but to determine whether a fully automated, TPS‐native workflow can produce prostate VMAT plans that are non‐inferior to clinically approved manual plans in terms of target coverage, OAR sparing, and deliverability. Within this context, the observed dosimetric differences should be interpreted primarily in terms of clinical equivalence, robustness, and consistency rather than absolute optimization of individual metrics. The following discussion interprets these findings in the context of existing automated planning approaches and considers their clinical implications and limitations.

Automated plans exhibited modestly higher monitor units and slightly lower modulation complexity scores compared with manual plans. This behavior is consistent with optimization strategies that prioritize systematic OAR sparing through iterative constraint relaxation rather than planner‐guided shape simplification.^29^, ^30^ Because the automated workflow applies the same feedback‐driven logic across all cases, it converges toward similar modulation patterns, whereas manual planning naturally reflects individual planner preferences and trade‐offs. From a clinical perspective, the observed increase in MU is unlikely to be meaningful. For prostate VMAT, the additional MU translates to a negligible increase in beam‐on time. Likewise, while lower MCS values nominally indicate greater modulation, delivery accuracy was not compromised, as confirmed by consistently high gamma pass rates in measurement‐based QA. Importantly, there is no evidence that the modest increase in modulation observed here would negatively affect treatment outcomes, particularly given the absence of differences in target coverage or high‐dose OAR metrics.

Figure 2 illustrates substantial variability in optimization time across cases, driven primarily by (i) automated grid‐size selection, which directly impacts optimization and calculation time, and (ii) the number of Phase 2 optimization loops. The latter depends on patient‐specific anatomy and the similarity between the Phase 1 fluence solution and the deliverable Phase 2 result. For cases 48 and 50, longer dose recalculation time arose because, after switching to a 2 mm grid and 3% statistical variance, automated post‐recalculation analysis determined that the rectum V60 Gy < 1%, which was previously satisfied with a coarser grid, was no longer met. The script then applied a minor rectum OAR objective adjustment and reoptimized at 2 mm; all of this additional work is counted under “Dose Recalculation” in Figure 2. Only 2/50 cases required reoptimization at the finer grid resolution.

The fact that all automated plans met mandatory constraints and that 92% also satisfied all optimal objectives indicates that the automated workflow reliably produces plans suitable for routine clinical use, even in anatomically challenging cases. A small number of cases did not meet one or more optimal (but not mandatory) planning constraints, which are discussed in the following. The isolated exceedance of the bladder V60 Gy criterion was attributable not to bladder volume, but to a uniquely challenging anatomical configuration in which the bladder was in direct contact with the anterior prostate surface over approximately half of its superior–inferior extent. Similarly, in the two cases exceeding the optimal bladder V48 Gy constraint, bladder shape and volume characteristics contributed to the elevated dose metrics. Importantly, even in this context, the automated workflow achieved bladder sparing comparable to, and in one instance superior to, manual planning. The elevated rectal dose metrics observed in the fourth case were likely driven by atypical rectal anatomy, with the rectal center deviating substantially from the patient midline across multiple axial slices. Despite these anatomical challenges, all rectal dose metrics remained well within mandatory clinical limits.

Taken together, these outlier cases highlight the influence of individual anatomy on achievable dose distributions while reinforcing the robustness of the APA. Even when optimal constraints were marginally exceeded; overall plan quality remained high, supporting the clinical reliability of the automated workflow. This behavior aligns with routine clinical decision‐making, where optimal objectives are knowingly relaxed to maintain clinically acceptable plans.

The non‐inferior dosimetric performance observed in this study suggests that such feedback‐driven automation can achieve clinical‐quality outcomes without reliance on external models or large training datasets as used in other studies.^20^, ^25^, ^26^, ^27^

Our gamma pass‐rates (≥ 99.8%) comfortably exceed institutional acceptance (≥ 95%); indicating that the higher degree of modulation introduced by automated objective tuning did not compromise delivery accuracy. The primary focus of this work is a planning study, aimed at comparing a fully automated planning solution with clinically approved manual plans in terms of dosimetric quality, and consistency. While delivery verification was not the central scientific question being addressed, we elected to include it on a randomly selected subset of cases as supportive validation to confirm that the automated workflow does not introduce deliverability issues. The ten plans selected for ArcCHECK measurement verification were drawn by simple random sampling from the full cohort of 50 automated plans. All plans in the cohort were generated using the same prescription, beam geometry, clinical protocol, and automated optimization strategy. This resulted in a homogeneous population with tightly clustered modulation complexity and MU metrics. In such a setting, a random subset is expected to be representative of the overall cohort. TG‐218 recommends plan‐specific measurement‐based QA as a standard clinical practice and does not define acceptable reduced sampling rates for automated planning pipelines.^35^ Our use of subset QA is therefore not intended to redefine or relax clinical QA standards, but rather to provide confirmatory evidence of deliverability within the scope of a planning comparison study. Given the consistently high gamma pass rates observed for all sampled plans, together with the narrow distribution of complexity metrics across the cohort, extending physical measurements to all 50 cases was unlikely to yield additional insight for the objectives of this study.

This study has several limitations that should be considered when interpreting the results. First, it represents a single‐institution experience, reflecting local clinical protocols, planning practices, and quality assurance procedures. While this controlled environment was intentional and appropriate for a non‐inferiority planning study, it may limit direct generalization to institutions with different planning objectives, TPS configurations, or clinical workflows. Variability in the manually generated plans reflects routine clinical practice, where planners are afforded flexibility in optimization strategy; this variability may have been reduced by enforcing a standardized planning protocol, which could in turn have narrowed differences in consistency between manual and automated plans.

Second, the evaluation was restricted to a single prescription and fractionation scheme (60 Gy in 20 fractions) and to single‐arc VMAT geometry. Although this regimen reflects a common contemporary prostate protocol, the performance of the APA for alternative fractionation schedules, prescriptions, or multi‐arc geometries was not assessed and warrants future investigation. Third, patients requiring non‐standard beam setup, such as patients with metal hip implants or bowel directly adjacent to the prostate were excluded, as these cases require a modified clinical planning strategy and a different automation workflow. The present results therefore apply to the majority of routine prostate cases but do not encompass all anatomic presentations encountered in clinical practice. A further limitation of this study is that plan evaluation was primarily based on DVH metrics, without a formalized, blinded qualitative review of dose distributions by experienced planners or clinicians; although all plans met clinical acceptability criteria, subtle spatial characteristics such as hot spot location, and dose gradient characteristics may not be fully captured by DVH‐based analysis alone.

With respect to delivery verification, clinical baseline plans were already delivered under routine institutional QA, and automated plans demonstrated robust TG‐218–level gamma pass rates. Manual planning time was not formally recorded, precluding a direct, end‐to‐end comparison of total clinical workflow time between manual and automated planning. Future studies should benchmark not only optimization time but also overall staff utilization and clinical throughput to more fully characterize operational benefits. Finally, this work was designed to assess dosimetric non‐inferiority and deliverability, rather than long‐term clinical outcomes. While equivalent target coverage and OAR sparing suggest comparable treatment quality, clinical endpoints such as toxicity and biochemical control were beyond the scope of this study.

## 5 CONCLUSION

In a retrospective, paired comparison against clinically approved manual plans, our TPS‐native, feedback‐driven Monaco automation for localized prostate VMAT—built entirely on existing hardware—demonstrated non‐inferior target coverage, meaningful reductions in low–mid rectal and bladder dose, and no loss of deliverability, with an execution time of approximately 40 min per case. By codifying expert adjustments and removing human‐in‐the‐loop variability, the workflow produced more consistent plan quality across the cohort. These findings support deployment of TPS‐resident automation as a practical means to standardize outcomes in everyday prostate planning.

## AUTHOR CONTRIBUTIONS

The authors contributed to the paper as follows: study design: Joel Sangster, Friedlieb Lorenz, David Jolly, Jerome Gastaldo; data analysis: Joel Sangster, Friedlieb Lorenz, Megan Taylor; measurement data collection: Joel Sangster, Megan Taylor; manuscript preparation and revision: Joel Sangster, Friedlieb Lorenz, Megan Taylor, David Jolly, Jerome Gastaldo.

All authors reviewed the results and approved the final version of the manuscript.

## CONFLICT OF INTEREST STATEMENT

The authors declare no conflicts of interest.

## ETHICS STATEMENT

The authors have nothing to report.

## Supporting information



Supporting Information

## Data Availability

Data used to generate the results in this manuscript is available upon reasonable request to the corresponding author. Full pseudocode of the APA is available in the supplementary material.
