# Supplementary material for: A rule‐based, feedback‐driven framework for fully automated prostate VMAT planning
Source: J Appl Clin Med Phys. 2026 Aug 24;27(9):e70751. doi: 10.1002/acm2.70751 (PMC13504152; doi:10.1002/acm2.70751)
Supplement: Supplementary file 1 [file ACM2-27-e70751-s001.pdf]

```
BEGIN PROGRAM

    Connect to Monaco application instance
    Initialise logging

    Minimise command prompt window

    TRY

        Set default dose calculation grid size = 0.49

        --------------------------------------------------
        LOAD PATIENT AND PREPARE STRUCTURES
        --------------------------------------------------

        Load patient
        Load selected study set

        Check bladder volume
        IF bladder volume is <170cc THEN
            Use finer dose calculation grid = 0.3
            Mark patient as "small bladder"
        ENDIF

        IF OptRectum structure does not exist THEN
            Create OptRectum using margins around Rectum: 2cm post, 0.3cm LR, clipped 0.5cm from patient surface
            Save patient
        ENDIF

        IF PenileBulb structure does not exist THEN
            Create ProstInf structure from PTV: ProstInf= (PTV + 2.5cm Inf) - (PTV + 4cm LR margin + 4cm AP margin)
            Map ProstInf to PenileBulb in IMRT Constraints
            Save patient
        ENDIF

        --------------------------------------------------
        CREATE PLAN
        --------------------------------------------------

        Create new plan from template

        Replace Rectum with OptRectum in IMRT constraints

        Determine and set isocentre position according to rules:
            X: IF ABS(Px-Cx) < 1cm THEN X = Cx ELSE X = Cx + ROUND(Px-Cx)
            Y: IF ABS(Py-Cy) < 1cm THEN Y = Cy ELSE {IF Cy + ROUND(Py-Cy) MOD 2mm == 0, THEN Y = Cy + ROUND(Py-Cy)  ELSE Y = Cy + ROUND(Py-Cy) + 1mm }
            Z: IF ABS(Pz-Cz) < 1cm THEN Z = Cz ELSE Z = Cz + ROUND(Pz-Cz)
        where (Cx,Cy,Cz) = CT ref coordinates, (Px,Py,Pz) = centre of PTV.

        Fill patient with ED = 0.8

        --------------------------------------------------
        INITIAL OPTIMISATION
        --------------------------------------------------

        Run optimisation phase 1

        Improve dose conformality using the 3 patient quadratic overdose constraints:
            1. 24Gy: IF IE < 0.5 THEN IC = IE * 1.3 ELSE IC = IE * 1.05
            2. Enable multicriterial (MCO) on 57Gy and 40Gy  patient QO
            3. Optimize Phase 1
            4. Modify patient QO:
                a. 57Gy: IF IE<0.025 THEN IC = 0.04 ELSE IC = IE *1.5
                b. 40Gy: IF IE<0.04  THEN IC = 0.1  ELSE IC = IE * 2.5
            5. Disable MCO on all patient QO

        Reset dose calculation engine
        Run optimisation phase 1

        --------------------------------------------------
        REDUCE OAR DOSES
        --------------------------------------------------

        Enable MCO for OptRectum serial constraint
        Run phase 1 optimisation
        Tighten OptRectum serial constraint: IC = IE + 5.2 Gy
        Disable MCO

        Enable MCO for Bladder serial constraint
        Run phase 1 optimisation
        Tighten bladder serial constraint: IC = IE + 6 Gy
        Disable MCO

        Tighten PenileBulb serial constraint: IC = IE

        --------------------------------------------------
        COVERAGE IMPROVEMENT LOOP
        --------------------------------------------------

        Define list of progressively smaller grid sizes: { 0.47, 0.45, 0.43, 0.4, 0.39, 0.38 }

        Reset dose calculation engine

        WHILE grid size options remain

            Run phase 1 optimisation

            IF PTV V57Gy < 95% OR CTV V60Gy < 96% THEN
                Reduce grid size
                Reset dose calculation engine
```

```
    ELSE
        Exit loop
    ENDIF

ENDWHILE

IF coverage still inadequate THEN
    Save and close patient
    EXIT PROGRAM
ENDIF

--------------------------------------------------
CHECK BLADDER AND PENILE BULB PRIORITIES
--------------------------------------------------

LOOP

    Find highest-weighted optimisation constraint

    Obtain bladder and penile bulb weights

    IF small bladder case THEN
        Relax constraints if penile bulb is the highest-weighted: IC = IE * 1.15
    ELSE
        Relax constraints if bladder OR penile bulb is the highest-weighted: IC = IE * 1.15
    ENDIF

    IF highest-weighted is not bladder or penile bulb THEN
        EXIT LOOP
    ENDIF

    Reset dose calculation engine
    Rerun phase 1 optimisation

END LOOP

--------------------------------------------------
COVERAGE REFINEMENT LOOP
--------------------------------------------------

Run phase 2 optimisation

Set iteration counter to zero

LOOP

    IF rectum V60 Gy > 0.88%  THEN
        Tighten rectum quadratic overdose: -= 0.03 Gy
    ENDIF

    IF bladder V60 Gy > 4% THEN
        Tighten bladder quadratic overdose: -= 0.08 Gy
    ENDIF

    Set PTV coverage tolerance to 95.9% and CTV coverage tolerance to 97%

    IF any rectum or bladder constraints out of tolerance
        Set PTV coverage tolerance to 95.1% and CTV coverage tolerance to 95.8%

    Calculate current CTV and PTV coverage

    IF coverage below tolerance AND iteration counter < 10 THEN
        IF rectum or bladder QO modified in this loop
            Ignore that QO in weights check
        ENDIF

        Determine the two highest weight in IMRT constraints

        IF both weights > 300 THEN
            Relax both cost functions
                IF affected cost function for penile bulb or patient
                    IC = IE * 1.8
                ELSE
                    IC = IE * 1.08
                ENDIF
        ELSE
            Relax only the highest weighted cost function
                IF affected cost function for penile bulb or patient
                    IC = IE * 1.8
                ELSE
                    IC = IE * 1.08
                ENDIF
        ENDIF

        Reset dose calculation engine
        Run batch optimisation

    ELSE
        IF rectum or bladder quadratic overdose modified in this loop
            Run phase 2 optimisation
        ELSE
            EXIT LOOP
        ENDIF
    ENDIF

ENDLOOP

--------------------------------------------------
FINAL DOSE CALCULATION
--------------------------------------------------
```

```
        Change grid to 0.2cm and variance to 3% per control point

        Run dose recalculation

        --------------------------------------------------
        FINAL QUALITY CHECKS
        --------------------------------------------------s

        Calculate:
            - CTV coverage > 95%
            - PTV coverage > 95%
            - Rectum V60 Gy < 1%
            - CTV D2% < 64.2

        IF any criteria fail THEN

            IF rectum V60 >= 1%
                Modify the rectum quadratic overdose: -= 0.03 Gy
            ENDIF

            Lower plan quality in sequencing parameters from 8 to 6

            Run batch optimisation again

            IF CTV coverage inadequate THEN
                Rescale 60 Gy to cover 95% of CTV
            ENDIF

            IF PTV coverage inadequate THEN
                Rescale 57 Gy to cover 95% of PTV
            ENDIF

        ENDIF

        --------------------------------------------------
        SAVE RESULTS
        --------------------------------------------------

        Save patient

        Export cumulative DVH to CSV

        Close patient

    CATCH exception

        Log error
        Close patient safely

    END TRY

END PROGRAM
```
